# Women's experience of preterm birth in an East African context: a qualitative study

**DOI:** 10.4314/ahs.v24i1.19

**Published:** 2024-03

**Authors:** Thomas Asmelash Habtu, Andrea Barnabas Pembe, Faraja Chiwanga, Jon Øyvind Odland, Elisabeth Darj

**Affiliations:** 1 Department of Public Health and Nursing, Norwegian University of Science and Technology, NTNU, Trondheim, Norway; 2 Department of Obstetrics and Gynaecology, Muhimbili University of Health, and Allied Science, MUHAS, Dar es Salaam, Tanzania; 3 Teaching, Research, and Consultancy Unit, TRCU, Muhimbili National Hospital, MNH, Dar es Salaam, Tanzania; 4 Department of Community Medicine, University of Tromsø, Tromsø, Norway; 5 International Research Laboratory for Reproductive Ecotoxicology, The National Research University Higher School of Economics, Moscow, Russia; 6 School of Health Systems and Public Health, Faculty of Health Sciences, University of Pretoria, Pretoria, South Africa; 7 Department of Women's and Children's Health, Uppsala University, Uppsala, Sweden

**Keywords:** Preterm birth, maternal experiences, Tanzania, Global health

## Abstract

**Background:**

Approximately 15 million children are born each year prematurely, representing more than 10 percent of all childbirths worldwide. Prematurity is an acute event and the leading cause of death among newborns and children under five. Sixty percent of these premature deaths occur in Sub-Saharan Africa and Southeast Asia.

**Objective:**

The current study aimed to explore and understand women's experiences and perceptions regarding giving birth prematurely at the National Hospital of Muhimbili in Dar es Salaam, Tanzania.

**Method:**

A qualitative method, using Interpretive Phenomenological Analysis approach was chosen to understand and describe the women's experiences. A semi-structured guide was used during the interviews. All interviews were audio-recorded and transcribed verbatim.

**Findings:**

Eight in-depth interviews were conducted. The analysis revealed three superordinate themes: (a) Emotional turmoil: unmet expectations shattering maternal identity, emotional distress, and loss of hope; (b) Adapting to preterm birth and challenges: the unexpected situation, lack of proper care, strenuous breastfeeding routines, and socioeconomic challenges; (c) Significance of proper care and emotional support: good maternal care, mother-to-mother and family support.

**Conclusion:**

This study provided a deeper understanding of women's experiences and perceptions of premature childbirth. The current study indicated the importance of caregivers' awareness of the women's emotional distress, their need to adapt to a sudden unexpected situation, and the necessity of emotional support.

## Introduction

The World Health Organization (WHO) states that the number of children born too early is increasing 1. There is a scarcity of knowledge regarding women's views on giving premature birth as significantly few qualitative studies, particularly from Low-and middle-income countries, have been conducted. The 2012 Global Action Report on Preterm Birth shows that over 60 percent of preterm births occur in Sub-Saharan Africa and Southeast Asia, which accounts for around 80% of the 1.1 million preterm birth-related deaths worldwide 2. Approximately 15 million children are born prematurely, before the 37^th^ week of gestational age, representing more than one in ten newborn children worldwide 1. Prematurity is a prevalent cause of death among newborns and children under five 3. According to WHO, three-quarters of these deaths are preventable by deploying current and cost-effective interventions 1. Moreover, preterm birth complications account for 30-50% increased mortality in early and mid-adulthood compared to those who are full-term born 4.

Known risk factors associated with preterm birth are extreme maternal age (<20 and >35 years), low social and economic status, anemia, use of herbal medicines, infections such as HIV, and premature rupture of the membranes 5. Children who survive might experience lifetime disabilities and complications with short- and long-term effects 6,7 Respiratory distress, immature brain and neurodevelopmental impairments, social-emotional problems, learning difficulties, and chronic diseases (e.g., heart disease, hypertension, and diabetes) are among the complications 3,6,8. Consequently, prematurity is a global challenge. A matched case-control study conducted at Bugando Medical Centre in Tanzania showed that several maternal factors increase the likelihood of premature deliveries 9.

Furthermore, several studies have reported mothers undesired psycho-emotional experience regarding preterm birth. According to Flacking *et al.*, 2012, mothers may experience guilt, worry, and sadness at the premature separation from their babies, which can have a negative impact on their mental health and ability to form a link with their offspring 10. Mothers may also experience emotions of powerlessness and loss of control, as well as a sense of exclusion from the care of their children 11.

The United Republic of Tanzania, with 59 million inhabitants, is a Lower-and-middle income country with an annual population growth rate of 3.1% 12. Approximately 45% of the Tanzanian population is under 15 years old (12). Tanzania's healthcare system comprises the public, social security, and private sectors. The government runs about 60% of Tanzania's healthcare facilities 13. The country's antenatal care coverage of at least four visits is 62%, while maternal mortality was in 2022, 524 per 100 000 live births, and neonatal mortality 20 per 1000 live births 14. Thus, it is challenging for women and neonates to receive essential healthcare.

The global alliance to prevent prematurity and stillbirth (GAPPS) is among many initiatives that seek to improve birth outcomes worldwide by reducing the burden of premature birth and stillbirths 15. GAPPS works to close the knowledge gap in understanding the causes of preterm birth and collaborates to implement evidence-based interventions to improve birth outcomes and accelerate research focused on discovering and developing interventions to prevent preterm birth 15. Moreover, Sustainable Development Goal 3 (SDG3), regarding “Good Health and Well-being,” is one of the 17 goals introduced by the United Nations in 2015 16. SDG3's target 3.1 intends to reduce maternal and child mortality and underlines the importance of minimizing the amount of preterm birth worldwide 16. The present study aims to explore and understand the phenomenon of women's preterm birth-giving experiences in an East African context.

## Methods

The current study has deployed a qualitative method with an Interpretative Phenomenological Analysis (IPA) approach for the research project. IPA's theoretical foundation originates from phenomenology and hermeneutics, which seeks to understand the subjects' perspective, unfolding the actual meaning of the lived experiences and exploring how people make sense of their experiences 17, 18. IPA is a beneficial approach for content analysis examining diverse, ambiguous, and emotionally driven subjects, which is essential for exploring and comprehending the lived experience, in this case, of women who gave birth to a premature child 17. This study focused on the criteria introduced in 2021 by Nizza et al. to promote quality in IPA through “constructing a compelling, unfolding narrative; developing a vigorous experiential and existential account; close analytic reading of participants' words; and attending to convergence and divergence” 19.

### Study setting

The research project study site is at Muhimbili National Hospital (MNH) in Dar es Salaam, the largest referral and teaching hospital in Tanzania. A study conducted in 2015 at MNH found that the prematurity prevalence was 17.9% 20. The hospital has 1500 beds and six units in the Department of Obstetrics and Gynecology. Each unit runs an outpatient clinic and admissions. A Postnatal Unit at the clinic provides healthcare and follow-up to women and premature children discharged from the Neonatal Unit 21. The Department of Pediatrics and Child Health has a Neonatal Unit that provides care to premature, low birth weight, and small for gestational age children, and a Kangaroo Care Unit (KCU). There is also a Neonatal Intensive Care Unit with ventilators and a facility for Continuous Positive Airway Pressure (CPAP) and oxygen. A newborn with no complications and a childbirth weight > /= 1500g will be discharged with its mother after confirming that breastfeeding is sufficient. However, a mother with a premature newborn with a complication or birth weight less than 1500g will be admitted to the Neonatal Unit or the KCU, providing training and support to gain weight. A woman with a premature child often stays in the hospital for weeks and must pay for consumable supplies and essential newborn items.

### Participants

Eligible participants rich in information were purposefully sampled and recruited with an inclusion criterion of mothers who had given birth to a preterm child and were admitted at MNH 22. This study did not include women who gave birth to a stillborn child or with congenital anomalies. The study participants were admitted to the KCU, Neonatal and Postnatal Unit at MNH, with a temporal window of 2-8 weeks after giving birth. This time window was purposely chosen to avoid recall bias and give women enough time to grasp the experience of giving care to a premature newborn. The research participants were between 18-32 years old, on average 24.3 years, and varied in educational level. The women had attended antenatal care 1-6 times before giving birth, and they had 1-3 children during the study period; they stayed at the hospital on average 31 days (Table 1).

**Table 1:** Demographic characteristics of women giving birth prematurely at Muhimbili National Hospital

N=8	n
Age	18-32 years
Education	
Primary school	2
Secondary school	4
College	2
Estimated gestational age at labor	
33-36	2
28-32	4
< 28	2
Antenatal care visits	
0	0
1-2	3
3-6	5
Number of children	
1-3	8
4-5	0
Length of stay at MNH	
2-4 weeks	6
5-6 weeks	1
7-8 weeks	1

### Data Collection

The participants had one week to respond whether they wanted to participate in the study. We invited sixteen women to the research, and twelve agreed to participate. However, four women were discharged before conducting the interview. The women who participated in the study had signed consent forms. The women were interviewed once between February and March 2022 for an average of 43 minutes (35-62 minutes) while they were admitted at MNH. The interviews were conducted in the participant's native language, Kiswahili, and translated to English by a bilingual local research assistant. A pilot-tested interview guide was used during the interviews. Topics were developed from relevant literature and supervisors' experience and were discussed with the women. The topics included how the women experienced the process of giving birth prematurely, how they experienced the follow-up from the health personnel, and the experiences/feelings when they met the child for the first time.

### Data analysis

After it was evident to the first author that new information was not emerging during the last interviews, the research team stopped data collection, and the first author performed the data analysis between March and April 2022. The analysis was performed manually to gain a substantial understanding of the IPA process and become intimate with the data. Afterwards, the coding process started with the first participant by generating meaning units using the Line-by-Line method. Meaning units were organized into descriptive meaning content, then central or emergent themes were developed. Through abstraction, a technique of identifying patterns between emergent themes proceeded to create main categories or superordinate themes 17,18. Furthermore, the superordinate themes were examined with the hermeneutic circle approach to maintain the ideographic meanings of individual themes with the sense of the whole transcript. The hermeneutic circle refers to establishing a holistic understanding of a text based on individual paragraphs 18. After finishing the first participant analysis, the first author had to bracket the understanding and findings from the analysis and continue with the remaining participant transcripts 18. Smith et al. describe bracketing as putting aside prior knowledge, values, beliefs, and experiences to accurately analyze and interpret an IPA study 17. Quotes from the participants interviews are added in the results to illustrate the super-ordinate and ordinate themes (Table 2).

**Table 2:** Super-ordinate themes and ordinate themes supported by participant quotes generated during data analysis

Super-ordinate themes	Ordinate themes	Participant quotes
Emotional turmoil	Unmet expectations shattering maternal identity	R3: “I was afraid. I had anxiety about why I was referred here because when I came from… we were about four ladies, and the three were told to remain. They had just given birth there, so I was wondering why me. I was worried, and I felt fear”
	Emotional distress	R5: “I was worried most of the time; what was my baby doing? Is she crying, or is something bad happening? Is she asleep?”
	Loss of hope	R2: “Will she grow up? Will she survive? The first question I asked was, “Will she survive, and will she grow? She is too little. If I know the cause, I know what to do to the next pregnancy that there is probably something that I am making a mistake that makes me give birth to a premature baby.”
Adapting to preterm birth and care challenges	The unexpected situation	R1: “I felt like a normal pain, probably because of childhood. I thought it was just a normal pain; I didn't know whether I was told that I was already in labor. So, they said they are calling for an ambulance so I can go to Muhimbili National Hospital”
	Lack of proper care	R4: “The afternoon shift that I found here did not cooperate very much. They did not give me good care, and they just ignored me, I was sitting there, and nobody was concerned when I was in that condition”
	Strenuous breastfeeding routines	R6: “It is hard managing the movement because it's not easy. Actually, I have to accept that the baby has to stay there because of the care”
	Socioeconomic challenges	R1: “I asked my husband whether it is okay for me to go to Muhimbili National Hospital”
Significance of proper care and support	Good maternal care	R2: “There is this nurse who is so understanding, and once you tell her about the baby, she reacts fast and helps you”
	Mother-to-mother and family support	R4: “At first, I was excited and even cried; I called my mom and said, ‘Now I can hold my baby, my baby is no longer in the machine, I can even breastfeed,’ I was excited”

Finally, superordinate themes from all participant transcripts were examined with each case to establish patterns. Field notes and memos have widened the understanding and sphere of women's experiences of giving birth prematurely. The first author analyzed and interpreted superordinate themes reflecting the research question. Moreover, the interpretations were discussed repeatedly with the research assistant and supervisors to consider and confirm the first author's reflexive thoughts toward the women's experiences 23, 24.

### Ethical approval

Before commencing the study, ethical approval was acquired from the National Health Research Ethics Committee in Tanzania and the Regional Committee for Medical and Health Research Ethics in Norway. Research clearance and data collection permits were obtained from MNH. Before any data was collected, an informed consent form was signed by all participants. Given the phenomenon studied, with in-depth and possibly sensitive questions, the collected material was considered rich, and the women spoke freely about the unexpected preterm births and their experiences at the hospital.

## Results

Eight women with premature children delivered at MNH were interviewed. The findings provided three superordinate themes, revealing various emotions and feelings shared by the women during the interviews. The participants expressed guilt, fear, sadness, stress, separation, thankfulness, relief, hope, and hopelessness.

### Theme one: Emotional turmoil

Although giving birth and caring for one's child is a presumed natural phenomenon and expectation, mothers who give birth prematurely are going through a turbulent emotional roller-coaster. The mothers' unmet expectation had caused a chain of traumatic experiences by provoking sadness, lack of excitement, and endless worries. The women in the study repeatedly expressed their fear and stress because of early separation and lack of information about where their children were. Preterm newborns were placed either in the Neonatal Intensive Care Unit (NICU) or a Neonatal Unit after delivery. The women could not follow their children and lacked control over the situation. The incident put women through unfulfilling experiences, constantly thinking about what might happen to their children. Mothers' taken-for-granted expectation of touching and holding their babies was shattered after giving birth. Instead, they found their babies in a critical situation where they couldn't help but feel overwhelmed. The mothers' identity as core caregivers to their babies was constantly affected, and they suffered due to early separation and their babies' critical conditions. The mothers felt responsible for the children, yet they could not adequately perform their motherly duties and lacked attachment to their premature newborns. The mothers expressed their moments of great sadness, frustration, and guilt, finding their newborns under challenging situations, triggering doubt and questioning their self-worth.

“I was worried most of the time; what was my baby doing? Is she crying, or is something bad happening? Is she asleep?” (R5).

“The baby had oxygen and other tubes; I didn't understand what they were. And then, when I asked, I was told my baby couldn't breathe on her own. I was very anxious seeing the baby” (R3).

“The problem is once I reach the room, I could sometimes find the baby is on radiation. I felt very bad when I saw my baby in that light, so I could go outside and cry” (R7).

The women described traumatic experiences that caused stress, sadness, and lack of happiness. The unmet expectations precipitated prolonged uncertainty and lack of hope leading to emotional distress. None of the women had expected their babies to be born prematurely. Their knowledge about pregnancy, normal childbirth, and understanding of being a mother to a healthy baby disappeared immediately after the information that they were giving birth prematurely. Going through the unanticipated situation with no preparedness devastated their belief in the survival of their premature child. They were vulnerable to emotional distress due to identity crises and a lack of attachment to their babies. They suffered fear and loss because of the prolonged event they had unpreparedly faced.

“I was afraid. I had anxiety about why I was referred here because when I came from… we were about four ladies, and the three were told to remain. They had just given birth there, so I was wondering why me. I was worried, and I felt fear” (R3).

A mother who gave birth to twin preterm babies expressed shock, confusion, and being in a nightmare, which had extended to painful grief and emotional distress after losing one of her babies.

“… but later, after five days, I was told to go and see there, the babies just stay there, it's a routine movement to go and see the babies and then after arriving there one of the babies has died. And then I asked the nurses which one because I was expecting the one, they told me earlier, ‘the first one will die, so I expected the boy to be dead, but not the girl. And then I was told that the girl was the one who was died, and then I said I was confused. I didn't feel very well, I just saw others giving birth, and they stayed with their children; why my children” (R4).

“Will she grow up? Will she survive? The first question I asked was, “Will she survive, and will she grow? She is too little. If I know the cause, I know what to do to the next pregnancy that there is probably something that I am making a mistake that makes me give birth to a premature baby.” (R2).

### Theme two: Adapting to preterm birth and care challenges

All women described giving birth to a preterm baby as frightening and unexpected. They were in a world of tension with unpredictably severe and life-altering occurrences, yet there was a ray of hope through a chain of referrals. The perceived shocking news that they were already in labor came as a surprise after feeling minor discomfort on an ordinary day. The women had to gather all their strength to withhold and affirm their personal beliefs to endure what would happen next. They had endless questions to grasp the reality of something they did not anticipate. A fragile and vulnerable newborn, challenging environment, complex premature care routines, and acute distress have tested the women's ability to adapt to an unexpected situation and to convey adaptation and tolerance to overcome the challenges of their early motherhood experience. Different factors influenced the women's ability to adapt to new life crises. Their religious belief and perception propounded both fear and gratitude to overcome the challenges.

“I should be thankful. If I talk about a negative thing about this baby, God will hear, and he will take it back” (R2).

“How can I manage to go to the operation [Cesarean section] while the baby is still very young? So, does it mean I need to give birth to a baby who hasn't yet completed the time? To a premature baby? How will it survive? Probably the baby would die; I accepted the operation but felt that perhaps the baby would not be alive” (R5).

“It is hard managing the movement because it's not easy. Actually, I have to accept that the baby has to stay there because of the care” (R6).

“I felt like a normal pain, probably because of childhood. I thought it was just a normal pain; I didn't know whether I was told that I was already in labor. So, they said they are calling for an ambulance so I can go to Muhimbili National Hospital” (R1).

The need for early mother-child attachment bond establishment inflames mothers' desire to hold and breastfeed their premature babies. Lack of experience in breastfeeding and physiological limitations challenged the new mothers' expectations to feed and create a motherly bond. Women in the study described breastfeeding as a challenging yet rewarding experience when they overcame the circumstances. However, most preterm children were inserted with nasogastric tubes to receive their nutrition for the first few weeks. Therefore, the mothers had to learn from the nurses how to pump breastmilk and feed their babies, and they had to visit and try to breastfeed their babies every three hours, affecting their sleep and rest. The nurses were perceived as a crucial source of knowledge to adapt and fulfil the task and support them to embrace their fear of holding their child because of their small size and attached medical accessories.

“Whenever I tried to pinch my milk, there was nothing that came, so I continued thinking like, how will it be” (R8).

“The baby is too small; I don't even know where to hold and where not to touch. That was the scary thing… the first day, the milk didn't come out, there were just some drops. The nurses insisted that I continue pumping, just give the baby whatever you have, so I continued that way, that I felt good, but I was still scared because the baby was too small” (R5).

Giving birth to a premature baby and receiving intensive care at the hospital had put families under substantial socioeconomic constraints. The respondents had expressed their worry and most profound concern about their future of having babies with demanding care. Most women who had formal work, small businesses, or participate in income-generating activities were uncertain about their future. They had to depend on family and partner support even in medical emergencies, and the length of the hospital stay was neither predicted nor confirmed. Therefore, the mothers are at risk of not maintaining their social network and flow of social support outside their close family.

“I asked my husband whether it is okay for me to go to Muhimbili National Hospital” (R1).

“Usually, when you are admitted for the first time, many people would come. Friends' relatives would come, but when time goes by, for example, after three weeks it will start decreasing in number, like only those closest to you will be coming, your mother, your husband, or your parents, it is not easy” (R6).

Some women admitted to the delivery and maternity unit expressed how they felt unseen and forgotten regarding their urgent need for information and proper healthcare. They felt ignored and experienced negligence, and they said that the healthcare providers did not show empathy or not paying attention to their health-related concerns.

“The afternoon shift that I found here did not cooperate very much. They did not give me good care, and they just ignored me, I was sitting there, and nobody was concerned when I was in that condition” (R4).

“I could not explain to the doctor other than my water is leaking, yet I don't have labor pain, but the doctor ignored me and went away” (R2).

### Theme three: Significance of proper care and support

Providing proper care and emotional support for the women was perceived as crucial for sustaining their psychological and physical well-being. All women expressed gratitude to those who provided guidance, encouragement, and emotional support and for sharing information about their children. This way of caring had a critical element in their ability to adapt and remain calm in challenging life crises. The mothers said they were motivated and felt better after moving to the KCU. Having the children close and being involved in caregiving gave the women hope to take future responsibility. Furthermore, they experienced getting help and support from their own mothers and sharing experiences with other women who were admitted to the hospital because of prematurity. The mothers of the study participants and family members were mentioned as essential for emotional and social support throughout their hospital stay. The new mothers talked about the enormous joy and excitement of holding their baby for the first time and sharing the special moment with their mothers, the new grandmothers.

“There is this nurse who is so understanding, and once you tell her about the baby, she reacts fast and helps you” (R2).

“When I arrived, when I was at the labor ward, there was this doctor that I do not remember his name, he was a male, he was so good, he had encouraged me by saying don't worry, you will give birth to a precious baby no matter what and we are trying our best and keep praying to your God” (R7).

“Especially information giving like during ward round, most of the time I get the feedback after the ward round. For example, when they pass through, they notice a new thing about my baby; they have changed the medication, probably from this to the other. They are good at giving me information” (R6).

“It's a good feeling to have the babies and take responsibility because now it tells me that I am a mother and I need to know everything about my baby. I need to know his behaviors, what the baby likes and dislikes, all that” (R8).

“At first, I was excited and even cried; I called my mom and said, ‘Now I can hold my baby, my baby is no longer in the machine, I can even breastfeed,’ I was excited” (R4).

## Discussion

The present study aimed to establish an overview and understanding of mothers' lived experiences of giving birth to a premature child at MNH in Dar es Salaam, Tanzania. Unprepared, women encountered a frightening and overwhelming event that required adaptation to the preterm birth and care routines, manifested with a layer of challenging steps. Various factors impacted the women's adaptation capability, including their faith and social integrity, which equipped them with hope and the ability to be resilient. Unlike daily stressors, women prepare for motherhood through an expected timeframe of approximately nine months. Heydarpour et al. describe individual and social elements as the primary factors that affect adaptation to the role of motherhood 25. This study supports Heydarpour et al.'s conclusion that this adaption can be promoted by helping women of preterm infants, empowering them, and increasing their self-efficacy to eliminate negative factors and emotions in a period of turbulent emotions 25. Suddenly stepping unprepared into a significant and unexpected crisis may have substantial consequences. Similar to an American study on preterm births, no women in the current study were expected to give birth too early. The study reported the traumatic impact of preterm birth on parents shattering their parental expectations and forcing them to live in a state of uncertainty for an extended period 26. The women's experiences of fear and prolonged uncertainty are consistent with that of Goncalves et al., who found that 70% of mothers who gave preterm birth feared for their baby's life 27.

The women's unmet anticipation of a normal pregnancy was suddenly altered to being in a highly technological environment with a premature child. The situation triggered a chain reaction of painful events, such as fear, despair, lack of excitement, guilt, feelings of lonesomeness or negligence, grief, and prolonged worry. The women's unrealized expectations contributed to their adjustment to challenges, which have been viewed as a deprivation of desirable things to which one feels entitled and negatively affects their identity by undermining mental health 28, 29. Preterm birth is a risk factor for Post-Partum Depression (PPD), and anxiety was an emotion the women in our study expressed 30. Therefore, it is possible to suspect that the Tanzanian women who participated in this study are susceptible and vulnerable to PPD due to their exposure to psycho-emotional trauma and distress. The current study supports evidence from Flacking et al. and Rowe et al. which reported undesired psycho-emotional experience in relation to preterm birth that mothers may experience guilt, worry, and sadness at the premature separation from their babies, which can have a negative impact on their mental health and ability to form a link with their offspring 10,11.

It was a presumption for the women that they would breastfeed their newborns after delivery. Despite the effort to adapt to the difficult circumstances, the limitations caused a feeling of guilt concerning their lack of ability and the deterioration of the preterm newborn's health, which is also described by other authors when women were unable to breastfeed after delivery 26, 31. However, the participating women had to adapt to the situation and the available health care for the child to survive. They expressed happiness to hold their child for the first time yet worried they could not provide what was expected. Another situation the women had to endure was the immediate physical separation from their newborns, which caused distress and anxiety that provoked a lack of attachment and emotional chaos.

The current study supports evidence from a cross-sectional study by Trumello et al., which underlined the importance of involving NICU nurses and clinicians to optimize the care for mothers immediately after giving preterm birth and during the infant's hospitalization, considering the psychological needs of mothers of preterm infants 32.

Given the financial strains associated with giving birth to a preterm child and obtaining acute and special care at the hospital, the new mothers had to depend on their families. The women worried about the high cost of care, similar to a study from Ghana by Lomotey et al. and a health economic review by Hodek et al., that the health care of a premature infant has significant financial implications for parents 33, 34. The extended length of stay in the hospital and prescription of drugs not covered by health insurance, if the family has one, are vital factors to the unpredicted costs. The women often must depend on their husband's or the extended family's willingness to obtain emergency medical care. A similar situation was previously reported in Tanzania by Pembe et al., which described mothers having to adhere to social norms and abide by family members' wish to grant financial support during medical emergencies 35.

The women in the current study expressed their hope based on religious beliefs or societal norms that they use as an adaptation and resilience mechanism. The women described praying and thanking God during both difficult and relieving moments, as well as having a fear of complaining about the situation, resembling a South African study by Sih *et al.*^36^. Mothers of preterm infants may cope differently by praying, holding the child, or accepting the situation, which is determined by the condition of their newborns being cared for at the Neonatal Unit. They also depend on the support and information they receive from the nurses and doctors. Women in the study experienced a gap between the expected and actual support that healthcare professionals provide. They reported different levels of satisfaction regarding information, care and emotional support they received at different departments of maternity care. However, nurses served as an essential source of knowledge for the mothers to adapt and fulfil the breastfeeding task. Lomotey et al. consider that nurses have a teaching role in supporting mothers^33^. WHO recommendations on interventions to improve preterm birth outcomes states that an appropriate standard of childbirth care should be available to the mother in a facility with a team of healthcare providers competent in recognizing and safely managing preterm birth. Furthermore, that safe care during labor and childbirth requires close monitoring of the mother and special care for managing preterm newborns 37.

The women in the study appreciated their stay at the KCU, where they increasingly dared to take responsibility for caring for their babies. A study from rural Tanzania supports this finding that having the infants nearby and more involvement in the caregiving process improved attachment and capabilities of breastfeeding 38. They also experienced receiving help and support from their own mothers and other mothers admitted to the hospital. Even though the Neonatal and Kangaroo Care Unit did not deploy mother-to-mother-care as a care plan, the results agree with other studies that mothers have received practical and emotional support from their mothers and relatives 32, 38, 39. Moreover, this study has uncovered the necessity and importance of giving adequate information, appropriate care, and recognizing women's feelings and providing emotional support during preterm birth in order to maintain their emotional, psychological, and physiological well-being.

## Conclusion

The global effort to reduce maternal, neonatal, and child mortality through several initiatives, recommendations, and SDG goals may not be achieved without understanding and intervening into women's emotional, psychological, and physical health concerning preterm birth. The findings of this study indicate the importance of awareness among the health providers of the turbulent situation a woman giving preterm birth occurs in and the various mixed emotions of fear, hope, loneliness, and guilt they may have. Further, new mothers try to adapt to the unexpected circumstances they suddenly find themselves in. The health system is supposed to provide optimal quality of care to reduce negative experiences after spontaneous premature delivery. Supporting distressed mothers emotionally and psychologically and providing information regarding the baby's health is necessary for the mothers to adapt to the unexpected situation. The expertise of nurses, midwives, and doctors have is valued and their role is essential in assisting women with preterm infants at MNH and similar low-resource settings.

## Limitations and strengths

The low number of participants in this study might be considered a limitation. However, there is an ongoing discussion regarding the sample size in qualitative studies. Sandelowski recommends that qualitative sample sizes should be large enough to allow the unfolding of a new and richly textured understanding of the phenomenon under investigation but small enough so that the deep case-oriented qualitative analysis is not precluded 40. For instance, qualitative studies by Pembe et al., in Tanzania, interviewed six women, Lometey et al., in Ghana, ten women; and Sih et al., in Cape Town, included eleven women in their studies^32^, ^35^, ^36^. Similarly, this study's data collection continued until the interviewer did not obtain any new information. Though probing for more, the material was regarded as saturated after these eight interviews, and no more women were included. Through these eight interviews, we were able to unfold new and rich material in depth. A strength of this study is the national and international collaboration with senior researchers with expertise in maternal and neonatal health and their ability to conduct qualitative research in Low-and-middle income countries. Moreover, this study has implemented the criteria introduced in 2021 by Nizza et al. to promote quality in an IPA study 19.

## Trustworthiness

The above strength ensured the Credibility of the study, together with the systematic analysis of the data, periodically revisiting the data, reflections, and illustrations of the content with quotes from the participants. Transferability was achieved by describing the context and setting where the study was conducted and the participating respondents in detail. Consistency was guaranteed by performing the study in a short period of time. Trustworthiness through these aspects was illustrated for the readers to consider if this study is relevant to their context, but there is no aim to generalize the results. The novelty in this study is the use of the IPA, which offers insights into how a given person, in a given context, makes sense of a given situation of personal significance and includes a significant life event.
